# Application of intentional reimplantation in the treatment of severe periodontitis

**DOI:** 10.1097/MD.0000000000046514

**Published:** 2025-12-26

**Authors:** Liye Song, Jiahuan He, Juanhua Chai, Yue Cui, Ying Guo, Xiaobo Zhang, Mengfei Yao

**Affiliations:** a Department of Stomatology, Affiliated Hospital, Hebei University of Engineering, Handan, Hebei, China; b Department of Stomatology, Handan Central Hospital, Handan, Hebei, China; c Department of Dermatology, Affiliated Hospital of Hebei University of Engineering, Handan, Hebei, China; d Department of Dermatology, Handan City Eye Hospital (The Third Hospital of Handan), Handan, Hebei, China.

**Keywords:** ankylosis, intentional reimplantation, periodontal healing, severe periodontitis, tooth survival

## Abstract

Severe periodontitis is a major cause of tooth loss, and the management of hopeless teeth remains a clinical challenge. Intentional reimplantation has been proposed as a conservative alternative to extraction and prosthetic replacement, but its clinical value in periodontitis-affected teeth has not been fully established. In this study, sixty patients with severe periodontitis, each contributing a single tooth, underwent intentional reimplantation and were followed for 18 months. Clinical examinations and radiographic assessments were performed at 3, 6, 9, 12, and 18 months after surgery. At the final follow-up, 53 teeth were successfully retained, corresponding to a survival rate of 88.3%, with cumulative survival rates of 100%, 96.7%, 95.0%, and 91.7% at 3, 6, 9, and 12 months, respectively. Among the retained teeth, 45 (85.0%) developed ankylosis, and the overall improvement rate in mobility reached 83.3%. Periodontal parameters, including probing depth and adjacent bone loss, were significantly improved compared with baseline values (*P* < .05). These findings indicate that intentional reimplantation is an effective approach for selected teeth with severe periodontitis, allowing for preservation of natural dentition and restoration of function. Although ankylosis frequently represents the predominant healing modality, the procedure remains clinically valuable and may serve as an alternative treatment when conventional periodontal therapy is inadequate.

## 1. Introduction

Apical periodontitis and severe periodontitis are among the leading causes of tooth extraction. Dental implants have become the primary option for the replacement of missing teeth, providing patients with a so-called “second dentition.” However, implants achieve osseointegration with the alveolar bone rather than attachment through a periodontal ligament. Consequently, the biomechanical environment differs from that of natural teeth, as implants lack the cushioning function of the periodontal ligament. This results in a chewing experience that is not fully comparable to that of natural dentition. Moreover, implants cannot fully reproduce the gingival contour and soft-tissue esthetics of natural teeth, particularly in the anterior region where the red–white harmony of natural dentition is difficult to restore.

Intentional reimplantation represents a conservative therapeutic alternative for teeth deemed untreatable by conventional approaches. This technique involves atraumatic extraction of the affected tooth, extraoral management including debridement and root canal treatment, followed by replantation into the original socket.^[1]^ For patients with severe periodontitis who are motivated to preserve their natural teeth, intentional reimplantation offers a valuable option. Compared with implant-supported or fixed prosthetic restorations, intentional reimplantation avoids immune rejection and the psychological discomfort of a foreign body, requires a shorter treatment period without an edentulous phase, is less invasive, more cost-effective, and does not compromise adjacent teeth. Importantly, even in cases of unfavorable prognosis, subsequent restorative options remain feasible, and the procedure may provide favorable conditions for future rehabilitation.

A systematic review by Grossman ^[2]^ indicated that intentional reimplantation is a reasonable choice in cases where single-crown implant placement is not feasible. However, to date, there has been no comprehensive statistical evaluation of clinical outcomes following intentional reimplantation in teeth affected by severe periodontitis. Furthermore, the biological mechanisms underlying healing after reimplantation and the prognostic factors influencing treatment success remain insufficiently understood. Therefore, the present study was designed to evaluate the clinical efficacy of intentional reimplantation in teeth with severe periodontitis, to explore potential healing patterns, and to provide evidence for refining the indications and contraindications of this procedure.

## 2. Materials and methods

### 2.1. Research target

This study was approved by the Ethics Committee of Handan Central Hospital. Sixty affected teeth (29 anterior and 31 posterior teeth, of which 7 were multi-rooted) of 60 patients (33 males and 27 females) with severe periodontitis who visited our hospital from December 2022 to December 2023 were selected for intentional dental reimplantation, with an age range of (33.27 ± 7.19) years. Postoperative follow-up was 18 months. All human participants provided written informed consent to participate in the study. This study was approved by the ethics committee of the Affiliated Hospital. All participants provided written informed consent before participation. Clinical trial number: 2023[K]0100.

The inclusion criteria were as follows: no systemic diseases or contraindications to periodontal surgery; the presence of a site where the probing depth (PD) of the affected tooth reached or exceeded the root apex and looseness of degree III; the presence of a neighboring tooth with looseness of less than degree II and a distance of <2 mm from the affected tooth; and the patient’s strong desire to retain the affected tooth. The exclusion criteria were as follows: the affected tooth is residual root, residual crown, or a desire for orthodontic treatment.

### 2.2. Treatment

The same experienced oral surgeon performed all the procedures to ensure operational consistency. Prior to patient enrollment, the operator underwent a calibration process involving 5 pilot cases to standardize surgical steps, extraction force, and reimplantation timing. During the study, adherence to the standardized protocol was periodically reviewed to maintain procedural uniformity. The patients used Sipay Gingival Solution (Xinqikang Pharmaceutical Co., Ltd, Guangzhou, China, State Pharmaceutical License Z65020012, about 3–5 mL/times, 3–5 times/day) with rinsing for 3 minutes before the operation to disinfect the operative area, and minimally invasive extraction of the affected teeth in the recipient area was conducted after local anesthesia.

Intentional tooth reimplantation was performed. The operative tooth was cleaned of granulation tissue and residual fine tartar using a curettage instrument, taking care to avoid a healthy apical periodontium. The upper and middle portions of the root canals were treated with ethylenediaminetetraacetic acid for 1 minute and rinsed thoroughly with copious amounts of saline to remove any chemical residues. The crown was wrapped in wet gauze, and the root canal treatment was completed in vitro using an ultrasonic filling technique to ensure that the root canal filling material exceeded the root apex to achieve an overfilling effect. The apical foramen was completely closed by trimming the excess cementum tip. The granulation tissue in the extraction socket was scraped off simultaneously. The extraction socket was trimmed and properly deepened with a high-speed split drill, trimmed and shaped, soft tissues were trimmed, rinsed with saline, and bleeding was stopped. The operated teeth were placed in the sockets, and the position was adjusted to fit the neighboring teeth to ensure that there was no occlusal high spot during light biting.

### 2.3. Postoperative follow-up

All patients were administered cephalin + metronidazole orally, 2 capsules/time, 3 times/day, for 5 to 7 days. If the patients’ postoperative reactions were severe, with obvious localized swelling and pain, they could be administered dexamethasone 5 mg intravenously, once/day, and Rehabilitation New Liquid Gargle, and they were not allowed to bite the affected teeth for 3 months. Postoperative follow-up at 3, 6, 9, 12, and 18 months, when the follow-up examination of the affected teeth loosened, taking apical films and periodontal examination, and oral prophylactic cleaning if necessary, strictly emphasize the need for plaque control. Periodontal fixation was removed at 9 months postoperatively, and looseness of the affected teeth was checked. If the tooth looseness was greater than degree I, periodontal fixation was continued.

### 2.4. Periodontal testing

The periodontal indices of the affected teeth were recorded by the same surgeon in the preoperative period and at 3, 6, 9, 12, and 18 months after the operation. The preservation of the affected teeth was recorded, and X-rays were taken to observe the height and density of the alveolar bone. Improvement in the looseness of the affected teeth was recorded, and periodontal probing was performed to record the probing depth, gingival recession (GR), bone loss (BL) on the adjacent surfaces of the affected teeth, and changes in tooth mobility (TM).^[3]^ Tooth looseness was judged based on the mobility of the teeth in the horizontal, vertical, and proximal-distal-medial directions. Normal teeth have some slight physiological mobility in the horizontal direction where looseness exists. When the teeth are shaken in the labiolingual direction using forceps, if the teeth are loosened more than the physiological mobility, the amplitude is within 1 mm, which is the first-degree loosening; the loosening amplitude of 1–2 mm is the second-degree loosening; and the loosening amplitude of more than 2 mm is a third-degree loosening. If the tooth loosens in the labiolingual direction without loosening in other directions, it is classified as first-degree loosening; loosening in the labiolingual, proximal, and distal-medial directions is classified as second-degree loosening; and significant loosening in all directions, including labiolingual, proximal-medial, distal-medial, and vertical, is classified as third-degree loosening.^[4]^

### 2.5. Statistical analysis

All statistical analyses were conducted using SPSS software, version 28.0 (IBM Corp., Armonk). Tooth mobility (looseness) was analyzed using the Wilcoxon signed-rank test for paired samples. Periodontal indices, including PD, GR, and BL, were initially assessed for normality using the Shapiro–Wilk test. Variables that followed a normal distribution were compared using the paired *t* test, whereas non-normally distributed variables were analyzed using the Wilcoxon signed-rank test. A 2-sided *P*-value of < .05 was considered to indicate statistical significance.

Post hoc analysis: the sample size of 60 patients was determined based on the number of eligible cases meeting the inclusion criteria during the study period. A post hoc power analysis was performed using the observed differences in PD (mean reduction of 2.48 ± 0.86 mm) between baseline and 18 months. With α = 0.05 and this effect size, the achieved statistical power (1–β) exceeded 0.90, indicating that the sample size was sufficient to detect clinically meaningful differences. Therefore, the current sample number is considered reasonable and statistically adequate for the study objectives.

## 3. Result

### 3.1. Basic patient information

A total of 60 patients with severe periodontitis were included in this study, comprising 33 males (55.0%) and 27 females (45.0%), with a mean age of 33.27 ± 7.19 years. The mean operative time was 34.55 ± 5.68 minutes. Each patient received a mean of 1.53 ± 0.50 trial reimplantations. Among the affected teeth, 29 were anterior teeth (48.3%) and 31 were posterior teeth (51.7%), including 7 multi-rooted teeth (11.7%). No significant differences in baseline demographic or clinical characteristics were observed within the study cohort. Detailed baseline data are presented in Table 1.

**Table 1:** Baseline characteristics of patients undergoing intentional reimplantation (n = 60).

Variables	Value
Sex, n (%)	
Male	33 (55.0)
Female	27 (45.0)
Age (yr), mean ± SD	33.27 ± 7.19
Operative time (min), mean ± SD	34.55 ± 5.68
Number of trial reimplantations, mean ± SD	1.53 ± 0.50
Tooth position, n (%)	
Anterior teeth	29 (48.3)
Posterior teeth	31 (51.7)
Multi-rooted teeth, n (%)	7 (11.7)

### 3.2. Retention rate

All 60 affected teeth were followed up for 18 months. During the follow-up period, 2 teeth were lost at 6 months, 1 tooth at 9 months, and 1 tooth at 18 months postoperatively. The cumulative retention rate was 100% at 3 months, 96.7% at 6 months, 95.2% at 9 months, 91.7% at 12 months, and 88.3% at 18 months, with 53 teeth successfully preserved at the final follow-up. Radiographic evaluations demonstrated no evidence of root resorption or loss of the periodontal ligament space at 3 and 6 months postoperatively. At 9 and 12 months, 25 patients exhibited increased alveolar bone height and density. By 18 months, 45 patients demonstrated complete disappearance of the periodontal ligament space, accompanied by replacement of resorbed root surfaces with alveolar bone, consistent with bone ankylosis. Clinically, periodontal parameters showed marked improvement. None of the preserved teeth presented with fistula formation, recurrent purulence, or tenderness to percussion. Among the retained teeth, 45 exhibited features of cemental ankylosis – characterized by the absence of physiologic mobility, a high-pitched percussion sound, and radiographic loss of the periodontal space – corresponding to an incidence of 85%.

### 3.3. Looseness and tooth retention

The degree of TM was assessed preoperatively and at 3, 6, 9, 12, and 18 months postoperatively. Compared with baseline, TM significantly improved at each follow-up (*P* < .05). At baseline, 43 teeth (71.7%) exhibited degree III mobility, while 17 teeth (28.3%) had degree II mobility. No teeth were stable (grade 0) or within degree I mobility. Following intentional reimplantation, progressive improvements were observed. At 3 months, 21 teeth (35.0%) showed reduced mobility, corresponding to an improvement rate of 9.3%. At 6 months, 33 teeth (55.0%) improved, with only 27 teeth remaining at degree III, yielding an improvement rate of 30.8%. By 9 months, 41 teeth (68.3%) demonstrated improved stability, and the proportion of teeth with degree III mobility decreased to 31.7%. At 12 months, 46 teeth (76.7%) showed improvement, and only 14 teeth (23.3%) remained at degree III. At the final 18-month follow-up, 53 teeth (88.3%) demonstrated improved stability, with only 7 teeth (11.7%) still presenting degree III mobility. These findings indicate that intentional reimplantation significantly enhanced tooth stability over time, with the overall improvement rate increasing from 9.3% at 3 months to 83.3% at 18 months (Table 2).

**Table 2:** Changes in TM at different follow-up time points (n = 60).

Time point	Grade 0 (no mobility)	Grade I	Grade II	Grade III	Improvement rate (%)
Preoperative	0 (0.0)	0 (0.0)	17 (28.3)	43 (71.7)	–
3 mo postoperative	0 (0.0)	2 (3.3)	19 (31.7)	39 (65.0)	9.3
6 mo postoperative	0 (0.0)	11 (18.3)	20 (33.3)	27 (45.0)	30.8
9 mo postoperative	1 (1.7)	19 (31.7)	18 (30.0)	19 (31.7)	55.8
12 mo postoperative	2 (3.3)	22 (36.7)	22 (36.7)	14 (23.3)	67.4
18 mo postoperative	4 (6.7)	21 (35.0)	22 (36.7)	7 (11.7)	83.3

### 3.4. Comparison of periodontal testing indices at different time points

Periodontal indices of the affected teeth were evaluated preoperatively and at 3, 6, 9, 12, and 18 months after intentional reimplantation. Compared with baseline, PD and BL significantly decreased at each follow-up, indicating marked periodontal improvement (*P* < .05). The reduction in PD was most pronounced within the first 3 months and then gradually stabilized. Gingival recession (GR) showed an initial increase after surgery, followed by a gradual decline after 3 months, but remained significantly higher than baseline values throughout the follow-up period (*P* < .05). Detailed results are presented in Table 3.

**Table 3:** Changes in periodontal indices at different follow-up time points (n = 60, mean ± SD).

Time	PD/mm	GR/mm	GR/mm
Preoperative	7.73 ± 0.85	1.67 ± 0.22	81.21 ± 1.76
3 mo after surgery	6.21 ± 0.77*	2.63 ± 0.17*	80.52 ± 2.21*
6 mo after surgery	5.62 ± 0.17*	2.59 ± 0.87*	79.57 ± 1.88*
9 mo after surgery	5.44 ± 0.45*	2.52 ± 0.35*	61.44 ± 3.26*
12 mo after surgery	5.31 ± 0.94*	2.44 ± 0.69*	59.24 ± 2.51*
18 mo after surgery	5.25 ± 0.46*	2.39 ± 0.41*	52.15 ± 4.87*

* indicates *P* < .05 compared with preoperative.

## 4. Discussion

Previous studies have demonstrated that the success of intentional reimplantation is influenced by several clinical factors, including the severity of periodontal disease and the presence of root fractures. Compared with dental implants, intentional reimplantation is less costly, technically less invasive, and associated with fewer postoperative complications.^[3]^ Nevertheless, insufficient control of periodontal inflammation remains a primary cause of reimplantation failure in patients with periodontitis, and for this reason, some authors have regarded periodontitis as a contraindication for this procedure. However, intentional reimplantation allows for direct removal of calculus and necrotic tissue from the alveolar socket and root surface, which has led to its increasing acceptance among periodontists. Other risk factors, such as smoking, age, number of deep periodontal pockets, and furcation involvement, have also been reported to affect treatment outcomes.^[4]^ Younger patients (<40 years) generally achieve better prognosis compared with older individuals, and the presence of multiple deep pockets has been associated with lower survival rates.^[5]^ Yoshizawa et al^[6]^ further reported that orthodontically extracted and cryopreserved teeth reimplanted after 18 months showed favorable periodontal healing and no reduction in alveolar ridge height, suggesting the feasibility of extended extraoral storage under controlled conditions.

Advances in periodontal regenerative techniques and improvements in root surface and alveolar management have progressively expanded the indications for intentional reimplantation. For teeth with combined periodontal–endodontic lesions, careful case selection is critical. Accurate identification of the primary etiologic source – whether periodontal, endodontic, or combined – is essential for determining treatment planning.^[7]^ Common features in reported cases include a poor response to conventional periodontal therapy and a strong patient preference for tooth preservation, often driven by economic concerns or the desire to avoid prosthetic replacement. In such cases, clinicians should carefully weigh the risks, benefits, and costs of different treatment modalities, with informed patient consent being an indispensable prerequisite.^[8]^

In the present study, intentional reimplantation was applied to teeth with severe periodontitis, which were otherwise considered hopeless. During the 18-month follow-up, significant improvements were observed in mobility, PD, and BL, while GR increased postoperatively but gradually stabilized. Overall, the affected teeth were preserved and regained functional capacity, demonstrating satisfactory clinical efficacy. Post-reimplantation outcomes can follow several healing pathways.^[9]^ Periodontal healing, which may occur through reattachment or new attachment, is the most desirable outcome and depends on the presence of viable periodontal ligament cells on the root surface and within the alveolar socket.^[10]^ Although rare in periodontitis-affected teeth, this healing mode has been occasionally reported.^[11]^ More commonly, healing occurs through bone ankylosis, particularly when the periodontal ligament is severely compromised. In this scenario, thorough debridement by mechanical and ultrasonic instrumentation facilitates close contact between the root surface and alveolar bone. Bone ankylosis is typically associated with replacement resorption, as described by Zhang et al,^[12]^ wherein osteoclast-mediated resorption of alveolar bone is followed by osteoblast-driven bone deposition that gradually replaces root dentin and cementum.^[13]^ Another potential complication is inflammatory resorption, which occurs when persistent inflammation triggers progressive root and alveolar bone destruction. If rapid, this process can lead to reimplantation failure.^[14]^ Although calcium hydroxide filling has been shown to reduce the incidence of inflammatory resorption, a large-scale study involving 838 reimplanted teeth reported an overall success rate of 88%, with root resorption accounting for 11% of failures.^[15]^ However, most cases in that study involved teeth with healthy periodontal status, underscoring the increased risk in periodontitis-affected teeth.

Moreover, previous studies have emphasized that the outcome of intentional reimplantation largely depends on the balance between periodontal ligament regeneration and replacement resorption. Periodontal healing is characterized by the reformation of a functional ligament space, preservation of physiologic mobility, and maintenance of root integrity, which can occur when viable periodontal ligament cells survive on both the root and alveolar socket surfaces.^[11,12]^ Conversely, when the periodontal ligament is severely destroyed, osteoclastic and osteoblastic activities lead to direct fusion between bone and root surfaces, resulting in replacement resorption or ankylosis.^[12,16]^ Although replacement resorption eliminates the periodontal space, it often ensures tooth stability and functional retention, which may represent a clinically acceptable outcome in cases of severe periodontitis.^[9,16]^ These findings suggest that while true periodontal healing is biologically ideal, replacement resorption may serve as a practical form of healing under compromised periodontal conditions.

To date, no systematic evaluation has been conducted specifically on intentional reimplantation in severe periodontitis. Given the poor baseline periodontal conditions of these teeth, inflammatory root resorption remains a complication that warrants close clinical attention. With continued advances in regenerative therapies, intentional reimplantation may serve as a viable adjunctive option for teeth unresponsive to conventional periodontal surgery. However, because the residual periodontal ligament in periodontitis-affected teeth is often limited, true periodontal healing is difficult to achieve. Therefore, optimizing surgical management of the root and alveolar socket to enhance stabilization, minimize resorption, and reduce recurrence of inflammation should represent an important focus for future research.

This study has several limitations. First, the sample size was relatively small, which may limit the statistical power and generalizability of the findings. Second, the follow-up period of 18 months was relatively short and may not fully reflect long-term outcomes such as progressive root resorption or late tooth loss. Third, the absence of a control group prevents direct comparison with conventional periodontal surgery or implant treatment. Additionally, all procedures were performed by a single operator in a single center, which ensured procedural consistency but may reduce external applicability. Future multicenter studies with larger sample sizes, longer observation periods, and appropriate control groups are needed to further validate these findings.

## 5. Conclusion

Intentional reimplantation can serve as an effective and conservative treatment option for selected teeth with severe periodontitis that are otherwise considered hopeless. The procedure achieved a high short-term survival rate and significant improvement in periodontal parameters, including PD, BL, and TM. Although replacement resorption and ankylosis were common healing patterns, most teeth remained functional and symptom-free during the follow-up period. These findings suggest that intentional reimplantation may provide a viable alternative for tooth preservation when conventional periodontal therapy is insufficient, particularly for patients with a strong desire to retain their natural dentition.

## Author contributions

**Conceptualization:** Liye Song, Jiahuan He, Juanhua Chai, Yue Cui, Ying Guo, Xiaobo Zhang, Mengfei Yao.

**Data curation:** Liye Song, Jiahuan He, Yue Cui, Ying Guo, Xiaobo Zhang, Mengfei Yao.

**Formal analysis:** Liye Song, Jiahuan He, Juanhua Chai, Yue Cui, Ying Guo, Mengfei Yao.

**Funding acquisition:** Jiahuan He.

**Investigation:** Jiahuan He, Xiaobo Zhang, Mengfei Yao.

**Writing – original draft:** Jiahuan He, Juanhua Chai, Ying Guo.

**Writing – review & editing:** Liye Song, Jiahuan He, Juanhua Chai.
